# A Complicated Case of Acute Promyelocytic Leukemia in the Second Trimester of Pregnancy Successfully Treated with All-*trans*-Retinoic Acid

**DOI:** 10.1155/2015/634252

**Published:** 2015-03-05

**Authors:** Kanika Agarwal, Megha Patel, Vandana Agarwal

**Affiliations:** ^1^The Commonwealth Medical College, 525 Pine Street, Scranton, PA 18510, USA; ^2^New Hope Cancer and Research Institute, 350 Vinton Avenue Suite 101, Pomona, CA 91767, USA

## Abstract

A 40-year-old female at 26-week gestation was diagnosed with acute promyelocytic leukemia (APL) after an abnormal prenatal lab workup showed pancytopenia. She was treated with all-*trans*-retinoic acid (ATRA), idarubicin, and dexamethasone. After day one of treatment, she developed differentiation syndrome, which was treated with dexamethasone. At 30-week gestation, she had preterm premature rupture of membranes and delivered by cesarean section because of the fetus' breech presentation. Despite ATRA's potential for teratogenicity, a viable infant was born without apparent anomalies. Postpartum, she underwent consolidation treatment with ATRA and arsenic trioxide (ATO). The patient continued ATRA therapy after delivery and is currently in remission.

## 1. Introduction

Acute promyelocytic leukemia (APL) is a variant of acute myeloid leukemia (AML) characterized by a translocation between chromosomes 15 and 17 [t(15;17)] [1]. APL in pregnancy is rare, with less than 60 cases described in the literature [2]. Its prevalence is expected to rise in developed countries due to the steadily increasing average maternal age of pregnancy [3]. All-*trans*-retinoic acid (ATRA) is seen to be an effective treatment of APL during pregnancy, resulting in remission in more than 90% of cases [4]. However, her treatment with ATRA is controversial because of its teratogenicity to the fetus. Despite this, we describe a patient with APL during pregnancy who was treated successfully with all-*trans*-retinoic acid (ATRA). She then suffered from differentiation syndrome as a side effect but still delivered a viable healthy infant.

## 2. Case Presentation

A 40-year-old gravida 4 para 3003, at 26-week gestation, was referred by her gynecologist to the obstetrics department due to abnormal prenatal lab results. Her recent bloodwork revealed pancytopenia with a white blood cell count of 3.3 × 10^3^/*μ*L, hemoglobin 9.7 g/dL, and platelet count of 29,000/*μ*L. The patient complained of easy bruising, increased fatigue, and low-grade fevers for 5 months duration. Further workup revealed a reticulocyte count of 4.33%, immature reticulocyte fraction of 0.62%, haptoglobin of <26 mg/dL, D-dimer > 5,250 ng/dL, and fibrinogen 97 mg/dL. Ultrasound showed a fetus at 26-week gestation with normal amniotic fluid index and normal fetal anatomy. Her past medical history, family history, surgical history, and OB/GYN history were insignificant. The patient denied smoking or alcohol consumption and had no history of prior radiation exposure.

**Figure 1:**
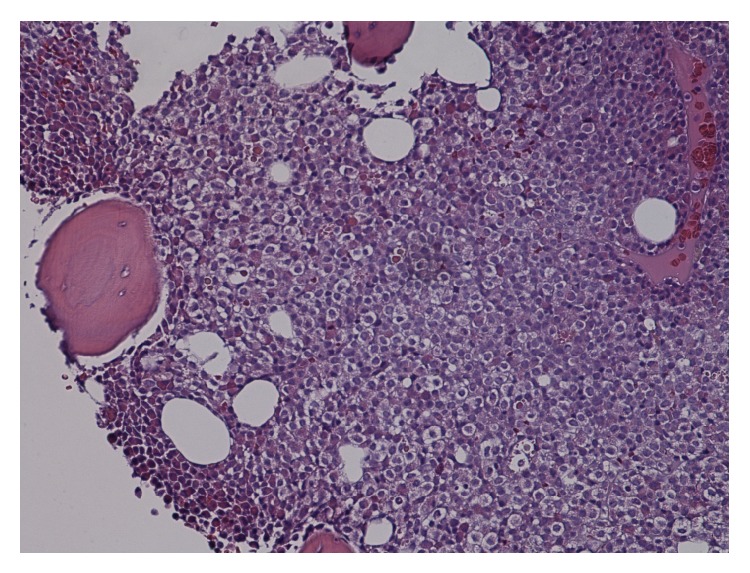
The patient's bone marrow biopsy revealed hypercellular bone marrow.

**Figure 2:**
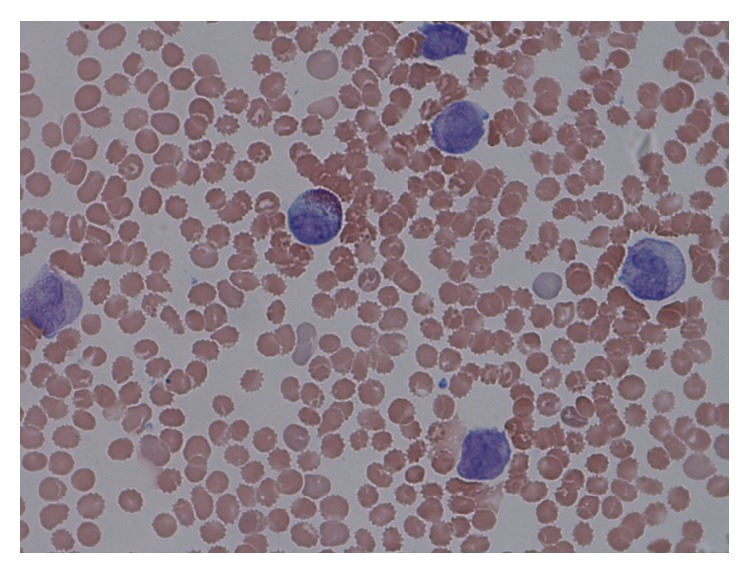
The patient's peripheral blood smear revealed promyelocytes containing Auer rods, which is consistent with APL.

A bone marrow biopsy revealed a hypercellular marrow with 90% promyelocytes containing Auer rods, along with scattered megakaryocytes and reticulocytes (Figures 1 and 2). Flow cytometry was performed to confirm the presence of myeloid blasts. Fluorescent in situ hybridization (FISH) analysis on the bone marrow showed PML-RAR*α* t(15,17) in 85% of the cells confirming acute promyelocytic leukemia. PCR was performed on the patient's bone marrow and confirmed PML-RAR*α* t(15,17). The elevated D-dimer and low fibrinogen levels were consistent with the diagnosis of concurrent disseminated intravascular coagulopathy (DIC).

The patient was started on all-*trans*-retinoic acid (ATRA) 45 mg/m^2^/day twice daily and idarubicin 12 mg/m^2^ every other day. Dexamethasone 10 mg twice daily was administered to prevent ATRA differentiation syndrome. The patient was also given transfusions of platelets and cryoprecipitate with goals of maintaining a platelet count of 100,000/*μ*L and fibrinogen levels of 200 mg/dL.

After one day of treatment, the patient developed hypotension, respiratory distress with hypoxemia, pulmonary edema, pericardial effusion, and transaminitis, suggestive of ATRA differentiation syndrome. She had a WBC count of 3.2 × 10^3^/*μ*L, hemoglobin 8.8 g/dL, and platelet count of 65,000/*μ*L. Her fibrinogen level was 251 mg/dL and had a D-dimer >5250 ng/dL. Her liver enzymes were elevated with her ALT at 107 U/L, AST at 131 U/L, and total bilirubin at 2.6 mg/dL. She was given albuterol nebulizer treatments and Lasix twice daily, in addition to continuing ATRA. Her dexamethasone therapy was adjusted to 4 mg every 6 hours for 10 days, which resulted in resolution of her differentiation syndrome. Upon resolution, her WBC count was 4.4 × 10^3^/*μ*L, hemoglobin 11.7 g/dL, and platelet count of 55,000/*μ*L. Her fibrinogen level was 305 mg/dL and she had a D-dimer >5250 ng/dL. Her liver enzymes returned to normal with her ALT at 33 U/L, AST at 23 U/L, and total bilirubin at 5.4 mg/dL Her respiration improved and she had decreased pericardial effusion as well upon resolution.

At 30 weeks of gestation, the patient had preterm premature rupture of membranes with the fetus in breech position. A Caesarian section was performed delivering a viable, healthy 3 lbs 2 oz female with no evidence of skeletal anomalies and APGAR scores 7 and 8 at 1 and 5 minutes, respectively.

A follow-up bone marrow biopsy 10 days postpartum revealed a hypercellular bone marrow (>80%) with myeloid hyperplasia and erythroid hypoplasia. Flow cytometry was negative for abnormal myeloid blast population with only 3% blasts, indicating complete remission. On postpartum day 15, the patient's labs revealed a white blood cell count 8.5 × 10^9^/L, hemoglobin 10.8 g/dL, platelet count 274,000/*μ*L, and fibrinogen 263 mg/dL.

She received consolidation treatment that began 17 days postpartum for 25 days. Her therapy consisted of ATRA 45 mg/m^2^ and arsenic trioxide (ATO) 0.15 mg/kg IV daily, which she tolerated well. In total, she received ATRA for 60 days since the start of her treatment. Prior to consolidation therapy, she had a WBC count of 6.5 × 10^3^/*μ*L, hemoglobin 4.68 g/dL, and platelet count of 294,000/*μ*L. Her PML/RAR*α* level was 0. After her consolidation therapy, PCR performed on her bone marrow could not detect PML-RAR*α* t(15,17), confirming she was in complete remission. After consolidation therapy, she had a WBC count of 1.3 × 10^3^/*μ*L, hemoglobin 8.7 g/dL, and platelet count of 29,000/*μ*L. Her PML/RAR*α* level was 0. She and the baby are doing well, as evidenced by the patient's negative FISH tests for t(15;17) four months postpartum. She is currently receiving another two additional cycles of consolidation treatment to ensure that she remains in remission.

## 3. Discussion

The hematologic manifestations of acute promyelocytic leukemia (APL), pancytopenia, disseminated intravascular coagulation, and hyperfibrinolysis represent a medical emergency during pregnancy. APL in pregnancy increases the risk of abortion, perinatal mortality, intrauterine growth retardation, and preterm delivery [5]. Clinical sequelae include increased bleeding, infection, inflammation, placental abruption, and decreased oxygen and nutrient delivery [6]. There have not been any other reports in the literature of such a presentation and complication during second trimester of pregnancy with a successful outcome, which makes our case unique.

APL is treated with all-*trans*-retinoic acid (ATRA) and anthracycline based chemotherapy, such as idarubicin or daunorubicin. It has a complete remission rate of greater than 90% and a potential cure in up to 80%. During pregnancy, this drug regimen is controversial due to the potential teratogenic effects and medication related complications like fatal retinoic acid syndrome [7]. Retinoic acid in low doses is especially harmful to the fetus during weeks 3 to 5 of gestation. Complications that can result include craniofacial alterations, neural tube defects, cardiovascular malformations, thymic aplasia, psychological impairments, and kidney alterations [4]. Miscarriages are estimated in 40% of patients with use of other retinoic acid derivatives during the first trimester of pregnancy [2]. However, there is a low risk of teratogenic effects during the second and third trimester of pregnancy. ATRA therapy was used in 27 cases, none of which resulted in congenital malformations in the newborn [4]. Reported fetal complications thus far from ATRA therapy include a successfully resuscitated fetal cardiac arrest and spontaneous resolved fetal arrhythmias [8]. Of those 27 cases, three resulted in spontaneous preterm delivery before 24 weeks [4]. In addition to fetal complications, ATRA can also cause maternal complications, such as differentiation syndrome. 24 of the 27 patients underwent complete remission, while the remaining three died postpartum [4].

Prior to the implementation of ATRA therapy, conventional chemotherapy was used for the treatment of APL during pregnancy. However, it is seen to trigger the release of procoagulants from promyelocytes, causing disseminated intravascular coagulation (DIC) to occur [9–12]. In patients whose ATRA is contraindicated, conventional chemotherapy can still be used but with certain risks. Receiving conventional chemotherapy during the first trimester can have teratogenic effects on the fetus during organogenesis (weeks 2–8), which include neural tube, heart, and limb defects. During the second and third trimesters, risks to the fetus include IUGR, low birth weight, and function of several organs [13].

Treatment with other chemotherapies, such as anthracycline antibiotics like idarubicin, reported no increase in teratogenic effects during the first trimester. A study with 28 women treated with combination chemotherapy including anthracycline antibiotics reported no fetal birth malformations. Of the 28, 24 had successful viable pregnancies with no complications, 2 experienced spontaneous abortions, and 2 resulted in fetomaternal demise [14].

While there are other therapies available to treat APL, ATRA is the most effective overall. In comparison to conventional chemotherapy, ATRA therapy improves DIC and prevents bone marrow aplasia from occurring [2, 4]. It is also seen to have a low risk of fetal malformations during the second and third trimesters [4]. ATRA is considered to be a better option in pregnancy due to the lower risk of teratogenic effects to the fetus.

Our patient's case of APL during pregnancy was further complicated by differentiation syndrome, also known as “retinoic acid syndrome.” Patients receiving ATRA treatment for APL have the risk of developing differentiation syndrome within 1–3 weeks of initiation of ATRA therapy [15]. This commonly presents with dyspnea, fever, weight gain, pulmonary infiltrates, or pleuropericardial effusion [15, 16]. Other characteristics of this condition may include hypotension, renal dysfunction, rash, and serositis [17].

Patients who develop differentiation syndrome from ATRA have a mortality rate of up to 30% if untreated due to hypoxemic respiratory failure or brain edema. Patients are treated with glucocorticoids and can show improvement within 12 hours or complete resolution within 24 hours. However, the mortality rate is up to 5% in patients treated with glucocorticoids [16, 18, 19].

The complete pathogenesis of differentiation syndrome has yet to be understood, but it has been thought to be due to the massive release of inflammatory vasoactive cytokines causing capillary leak, fever, edema, rash, and hypotension [20]. It is also thought that ATRA/ATO induces the maturation of promyelocytes, followed by the mature cells invading tissues [21].

Since there is limited information and reports about APL during pregnancy, it is undetermined whether the risk of differentiation syndrome is more or less likely during pregnancy in patients with APL receiving ATRA therapy [22]. However, a high WBC count at diagnosis has been associated with an increased incidence of differentiation syndrome [15]. In nongravid patients, the incidence varies from 2 to 27% in patients receiving standard doses of ATRA [15]. Patients receiving ATRA or ATO as maintenance or consolidation therapy for APL typically do not develop differentiation syndrome [17].

## 4. Conclusion

APL occurring during pregnancy is rare and the literature on the outcomes of these patients is limited, with less than 60 cases described [2]. Our patient's case is unique because of her presentation in pregnancy and further complication of differentiation syndrome. While receiving ATRA and suffering from differentiation syndrome, she gave birth to a healthy infant with no deformities. The patient has remained in remission with no other complications or adverse events since then. She will receive two additional cycles of her consolidation treatment to maintain her remission.

While successful treatment with ATRA has been documented in gravid patients, the literature is scarce for patients who experience complications from this treatment regimen. Our case is an example of a patient with a successful outcome despite complications that affect both mother and fetus. This would not have been achieved without the clinical management by a multidisciplinary team including a hematologist, oncologist, obstetrician, and neonatologist.
